# Alcohol and mental health care integration in traumatically injured patients with elevated BAC: a retrospective chart review

**DOI:** 10.1186/s13011-026-00736-3

**Published:** 2026-05-20

**Authors:** Nicholas R. Schumann, Madeline R. Marks, Devi Jayan, Jorian Greenwood, Sydney C. Timmer-Murillo, Timothy J. Geier, Claire M. Bird, Andrew T. Schramm, Sacha A. McBain

**Affiliations:** 1 Department of Trauma, The Queen’s Medical Center, Honolulu, HI USA; 2 Department of Psychiatry, University of Maryland, Baltimore, MD USA; 3 Department of Psychiatry and Behavioral Neuroscience, University of Chicago, Chicago, IL USA; 4 Division of Trauma & Acute Care Surgery, Medical College of Wisconsin, Milwaukee, WI USA; 5 Baylor Scott and White Research Institute, Baylor University Medical Center, Dallas, TX USA; 6 Department of Psychiatry and Behavioral Sciences, Rush University Medical Center, Chicago, IL USA

**Keywords:** Traumatic-injury, Alcohol use, Motor Vehicle Accidents (MVCs)

## Abstract

**Background:**

Alcohol is a well-documented risk factor for motor vehicle collisions (MVCs) and may also heighten post-trauma psychological symptoms. Given the frequent co-occurrence of MVC injury, alcohol use, and post-injury mental health sequelae, coordinated post-injury care is critical to support long-term psychological and physical recovery. The American College of Surgeons Committee on Trauma requires screening for and intervention on risky alcohol use and, more recently, to conduct mental health risk screening and referral. However, little is known about how established alcohol screening and intervention workflows interface with mental health services in routine practice.

**Methods:**

We conducted a retrospective cohort study of adults (≥ 18 years) with traumatic injuries at a Level I Trauma Center in the U.S. Midwest from 2017 to 2022. Data were pulled from the trauma registry and electronic medical record. A chart review was completed on a subset of patients who were legally intoxicated at the time of admission to assess engagement with services using a structured abstraction template.

**Results:**

From January 2017 to December 2022, 198 patients were admitted after MVC. Of the 154 patients tested for blood alcohol concentration (BAC), 78% were legally intoxicated (≥ 0.08 g/dL). 58% screened at high risk for PTSD or depression on the Injured Trauma Survivor Screen (ITSS). Of the patients legally intoxicated, 25 received at least one visit from trauma psychology. Alcohol use was discussed in 60% of these encounters, primarily regarding frequency and quantity. Eleven patients (45.8%) had alcohol use explicitly addressed and were provided resources by social work.

**Conclusions:**

These findings suggest that alcohol use, despite its significance in this high-risk population, was minimally addressed across specialty services.

## Background

Each year, an estimated 2.6 million patients are hospitalized for traumatic injuries in the United States, with alcohol contributing up to 62% of cases [15].

Motor vehicle collisions (MVCs) following intoxication are a particularly common alcohol-related injury [26], with harmful alcohol use increasing risk for rehospitalization [18]. Addressing alcohol use is not only a public health priority, it is a clinical imperative to reduce preventable injuries and improve long-term recovery outcomes [3]. Yet, sole focus on the intersection of alcohol use and the preventable physical injuries alone is insufficient for improving recovery-oriented outcomes [19].

Comprehensive prevention and recovery requires attention to the psychological consequences that parallel physical injury [11].

MVC survivors are at elevated risk for psychiatric morbidity, with 20–40% developing posttraumatic stress disorder (PTSD), major depressive disorder, or both [21, 30]. Population-based studies suggest that mental health concerns and substance use disorders cumulatively increase the risk for recurrent hospitalization and mortality [24, 28], increasing the likelihood of both initial and repeated trauma exposure, disrupting the natural recovery process, and compounding vulnerability to PTSD and ongoing alcohol use [28]. Existing evidence supports the effectiveness of interventions (e.g., motivational interviewing) focused on alcohol use in reducing re-injury and psychological interventions in improving post-injury health outcomes (American College of Surgeons, [1, 10]). Considering the comorbidity of MVC-related injury, alcohol use, and post-injury psychological sequelae, there is a need for coordinated post-injury care approaches to improve patients’ long-term psychological and physical recovery.

The American College of Surgeons Committee on Trauma (COT) requires trauma centers to screen for and intervene on risky alcohol use and, more recently, to conduct mental health risk screening and referral [2]. Some trauma centers have utilized existing alcohol screening and referral protocols as a model for implementation of post-trauma mental health risk screening and referral protocols [14, 16]. However, little is known about how established alcohol screening and intervention programs interface with integrated psychological services in routine practice at trauma centers. Variability across trauma centers in implementing these requirements, combined with staggered mandates and siloed clinical oversight, may create gaps and fragmented care for trauma patients with co-occurring harmful alcohol use and mental health risk.

This study aims to characterize existing inpatient alcohol related intervention practices for motor vehicle crash patients who presented with legal intoxication at admission. The study also aims to examine how these parallel protocols function in an institution with established independent alcohol and post-trauma mental health screening and intervention programs. We conducted a retrospective chart review of trauma patients in MVCs whose blood alcohol concentration (BAC) indicated legal intoxication (≥ 0.08 g/dL) [25]. Frequency of mental health screening and consultation, type of interventions delivered, and how alcohol use was addressed during admission were examined. By evaluating both access to and integration of care, this work aimed to identify opportunities to strengthen coordination of alcohol use- and mental health-related care for injured trauma survivors.

## Methods

### Setting

This study was conducted at a Level 1 Trauma Center in the Midwestern United States. The hospital maintains an in-hospital integrated psychological service staffed by licensed clinical psychologists, post-doctoral fellows, and supervised advanced graduate students. The service provides bedside psychological assessment and intervention (e.g., brief cognitive-behavioral interventions) in the acute injury phase. Consults were initiated by automatic referral triggers (i.e., positive mental health screen on the Injured Trauma Survivor Screen [ITSS]; [12]) or by request from the trauma surgery team. The ITSS is a nine-item yes/no screening tool used to identify potential risk for PTSD and major depressive episodes; a score of two or more indicates high risk. All patients receive standard trauma medical care, and the consult service functions as an adjunct intervention. The hospital-based social work service’s primary function is to support discharge planning, with additional clinical responsibilities that include conducting alcohol-use behavior screening, brief intervention, and referral to treatment (SBIRT) following a positive screen.

### Study design and population

This retrospective cohort study included adults ≥ 18 years old with traumatic injuries who were admitted to the hospital between 2017 and 2022. Data were obtained from the institution’s trauma registry and Electronic Medical Record (EMR). From the registry (*n* = 2,864), we identified an eligible cohort of patients who met the following inclusion criteria: (1) age 18-years or older, (2) Glasgow Coma Scale (GCS) score > 13 upon admission, (3) MVC as the mechanism of injury, and (4) reported BAC value. Patients were excluded if they expired during their admission.

The study protocol was approved by Medical College of Wisconsin’s Institutional Review Board (IRB #PRO00047211). A waiver of consent was obtained as all data was collected as a part of a retrospective chart review.

### Demographic and injury characteristics

Demographic characteristics included age, sex, and race/ethnicity. Injury characteristics included the injury severity score (ISS), and length of hospital stay. Descriptive statistics were used to characterize the sample.

### Chart review

Among the patients who were legally intoxicated (*N* = 154), a retrospective chart review was conducted for the subsample of individuals who received a trauma psychology consultation (*n* = 25). Reviewers abstracted data on alcohol-related interventions and psychological interventions documented during integrated psychology visits, as well as in clinical interactions with other services (e.g., social work, psychiatry). The chart review process involved the development of a structured abstraction template by the research team, data abstraction by a trained research assistant who was not involved in clinical care, and an iterative review of the extracted data by two clinical researchers with experience in qualitative methods and chart review [22].

### Domains of review

#### Integrated psychology service

For each psychology visit, we coded (1) alcohol-related themes (e.g., alcohol use disorder references, alcohol-related psychoeducation, withdrawal symptoms, motivational interviewing, harm reduction, abstinence), (2) psychological interventions targeting posttraumatic stress symptoms, depression, grief, adjustment, or emotional processing, and (3) the number and timing of follow-up visits. Each visit was then coded for its primary intervention target: alcohol-related concerns, post-trauma psychological distress, or concurrent focus on alcohol and psychological distress.

#### Multidisciplinary visits

We also reviewed documentation of patient engagement with other professionals (i.e., social work, psychiatry) regarding alcohol use and related concerns Coding included documentation of withdrawal protocols (e.g., Clinical Institute Withdrawal Assessment for Alcohol; [23]), medical management for alcohol withdrawal, completion of SBIRT protocol, and provision of resources for alcohol use treatment.

## Results

Our sample revealed that 154 (77.8%) patients were legally intoxicated (≥ 0.08 g/dL) according to state statute (*M* = 0.207; *SD* = 0.076; range 0.080–0.439). Table 1 describes the personal, injury, and mental health characteristics of those who received medical treatment for the MVC-related injury at the Level 1 Trauma Center. Characteristics are stratified by legal intoxication status. Table 2 displays the results from the patients of whom the chart review was completed.

**Table 1:** Personal, injury, and mental health characteristics (*N* = 198)

Characteristics	Legally Intoxicated *n* = 154	Below Legal Intoxication *n* = 44
**Personal**		
Age		
Mean (SD)	40.12 (14.38)	40.05 (16.13)
Sex, n(%)		
Male	112 (72.73%)	32 (72.73%)
Female	42 (27.27%)	12 (27.27)
Race, n(%)		
Black	70 (45.45%)	22 (50.00%)
White	63 (40.91%)	17 (38.64%)
Asian	4 (2.60%)	1 (2.27%)
Not Reported	17 (11.04%)	4 (9.09%)
Ethnicity, n(%)		
Not Hispanic	129 (83.77%)	40 (90.91%)
Hispanic	23 (14.94%)	3 (6.82%)
Not Reported	2 (1.30%)	1 (2.27%)
Blood Alcohol Concentration		
Mean (SD), range	0.207 (0.076), 0.080–0.439	0.028 (0.023), 0.010–0.076
**Injury**		
Total Hospital Length of Stay (days)		
Mean (SD), median	8.15 (12.97), 12.97	7.34 (9.53)
Intensive Care Unit, n(%)		
Yes	70 (45.45%)	25 (56.82%)
No	84 (54.55%)	19 (43.18%)
Length of Stay days yes ICU (Mean, SD), median	6.36 (8.40), 3.00	4.52 (3.72), 3.00
Injury Severity Score		
Mean (SD), range	15.22 (11.72), 1–75	14.61 (10.01), 1–42
**Mental Health**		
ITSS		
Screened		
Yes, n(%)	97 (77.60%)	24 (64.86%)
No, n(%)	28 (22.40%)	13 (35.14%)
* Results*,* Yes Screened*		
Positive PTSD, n(%)	8 (8.25%)	2 (8.33%)
Positive Depression, n(%)	16 (16.49%)	5 (20.83%)
Positive PTSD and Depression, n(%)	25 (25.77%)	7 (29.17%)
Risk Negative, n(%)	48 (49.48%)	10 (41.67%)
Number of Days from Injury to ITSS		
Mean (SD), median	2.505 (4.461), 1.00	2.625 (2.018), 2.00
Psychology Consultation		
Yes, n(%)	25 (16.23%)	6 (13.64%)
No, n(%)	129 (83.77%)	38 (86.36%)
Number of Days from injury to consult		
Mean (SD), median	5.760 (5.790), 3.00	3.167 (1.169), 3.00

**Table 2:** Personal, injury, and mental health characteristics for chart review: patients with BAC above the legal limit who received a trauma psychology consultation

Characteristics	*n* = 25
**Personal**	
Age	
Mean (SD)	38.28 (13.49)
Sex, n(%)	
Male	16 (64.00%)
Female	9 (36.00%)
Race, n(%)	
Black	9 (36.00%)
White	11 (44.00%)
Not Reported	5 (20.00%)
Ethnicity, n(%)	
Not Hispanic	19 (76.00%)
Hispanic	4 (16.00%)
Not Reported	2 (8.00%)
**Injury**	
Total Hospital Length of Stay (days)	
Mean (SD), median	17.36 (23.03), 12.00
Intensive Care Unit, n(%)	
Yes	14 (56.00%)
No	11 (44.00%)
Length of Stay days yes ICU (Mean, SD), median	10.07 (11.79), 4.00
Injury Severity Score	
Mean (SD), range	21.36 (12.21), 4–50
**Mental Health**	
ITSS	
* Screened*	
Yes, n(%)	18 (78.26%)
No, n(%)	5 (21.74%)
Missing	2
* Results*,* Yes Screened*	
Positive PTSD, n(%)	1 (5.56%)
Positive Depression, n(%)	2 (11.11%)
Positive PTSD and Depression, n(%)	11 (61.11%)
Risk Negative, n(%)	4 (22.22%)
Number of Days from Injury to ITSS	
Mean (SD), median	4.667 (9.081), 1.00
Number of Days from Injury to Consult	
Mean (SD), median	5.760 (5.790), 3.00

**Fig. 1:**
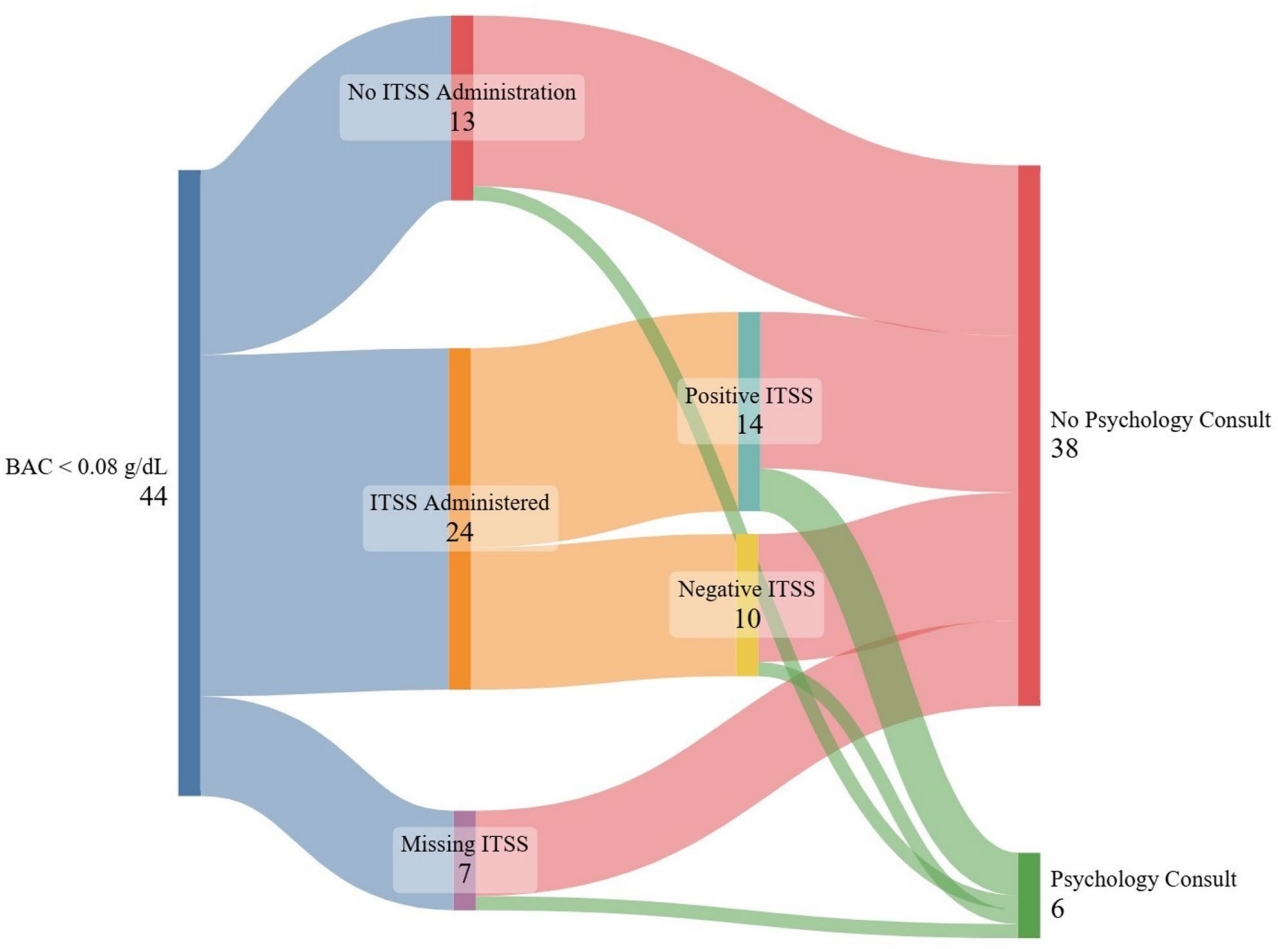
Sankey diagram of ITSS screening and consultation patterns for patients with BAL < 0.08 g/dL

See Fig. 1 for an overview of ITSS screening administration and consultation patterns.

### Content analysis of psychology consultation visits

#### Integrated psychology initial and follow-up visits

A total of 25 patients received an initial evaluation. Consults were primarily identified through a positive ITSS (*n* = 14) followed by referral from the trauma team (*n* = 11). The average duration was 26.6 min with a range of 5–50 min. Duration of visits was guided by patient or environmental factors (e.g., declining services, engagement, interruptions). A total of 15 (60%) unique patients had documentation of any alcohol-related assessment or intervention during an initial or follow-up visit. When alcohol was discussed, it included psychoeducation (*n =* 2), assessment of alcohol use (*n* = 9), and/or past or current outpatient alcohol use treatment (*n* = 4). A total of 5 patients had a pre-existing history of AUD documented by the trauma psychologist.

The primary focus of the intervention documented was adjustment to injury (*n* = 25). A subset of initial visits also documented intervention related to PTSD (*n* = 5), depression (*n* = 4), traumatic grief (*n* = 2), psychoeducation (*n* = 3), and emotional processing (*n* = 2). Harmful alcohol use was not documented as a primary focus of intervention in any initial consultations.

A total of 14 patients (56%) received at least one follow-up visit, with follow-up visits ranging from 1 to 9. A total of 3 (21%) had documentation of intervention related to alcohol use or withdrawal in a psychology follow-up visit. When alcohol use or treatment was documented, it included psychoeducation about alcohol dependence (*n* = 1), patient-reported treatment goals related to discontinuing alcohol use (*n* = 1), past treatment or treatment referral (*n* = 2), and documentation of alcohol use disorder (*n* = 2).

Psychological intervention in follow-up visits continued to be primarily focused on adjustment (*n* = 10), along with intervention focused on depression (*n* = 1), grief and/or emotional processing (*n* = 3), and reference to specific emotional coping strategies (*n* = 3).

#### Social work and psychiatry visits during admission

Among the 24 patients with social work involvement, initial contact typically occurred within 1–3 days of admission, with the number of follow-up visits ranging from 2 to 50. Approximately half of patients (*n =* 13) had no documentation of alcohol-related concerns or intervention. Eleven patients (45.8%) had alcohol explicitly addressed and received resources, most often through the SBIRT protocol (*n* = 9), though one declined SBIRT and another received a formal inpatient consultation with a substance use counselor. In this one case, pharmacotherapy (naltrexone) was also discussed.

Psychiatry consults were placed for six patients (24%) with visits primarily focused on withdrawal management in the context of AUD. Four patients received psychotropic medication management during admission, and two patients declined initiation of medication for withdrawal management. A total of 11 (45.8%) patients had a documented CIWA protocol during admission.

## Discussion

The aim of the current study was to explore inpatient alcohol and psychological intervention for MVC-injured patients who were legally intoxicated at the time of admission. In this cohort of patients admitted after MVC over five years, 78% of those tested for BAC were legally intoxicated. Given the low base rate of BAC being performed among MVC-injured patients, these results suggest that BAC was conducted in certain clinical contexts and is not representative of intoxication in the MVC trauma population at-large. Post-injury mental health risk was also common. Of the total sample, half of patients screened positive on the ITSS, including all 154 patients whose BAC indicated they were intoxicated These findings indicate a substantial co-occurrence of alcohol use and psychological risk in the study sample. The ITSS was not administered to 41 patients, most of whom were intoxicated, suggesting that acute intoxication may limit timely mental health assessment and a gap in the screening protocol for post-intoxication.

Analyses focused on the subset of legally intoxicated MVC patients who also received an inpatient trauma psychology consult, limiting the chart review to a small subset of patients. For these patients, per documentation, social work primarily focused on case management, trauma psychology concentrated on acute psychological adjustment to hospitalization and the secondary sequelae of MVC (e.g., traumatic grief, adjustment difficulties, acquired disability), and psychiatry emphasized medication management for AUD and withdrawal.

In examining alcohol-related interventions, the psychology service addressed alcohol use in 60% of cases, primarily assessing alcohol frequency and quantity, with two patients receiving documented psychoeducation on alcohol-related post-injury mental health risks. Similarly, alcohol screening and intervention were minimally represented in social work documentation.

Less than half of the sample had documented alcohol screening, and only about one-third had SBIRT administration recorded by social work. Psychiatry involvement largely focused on managing acute withdrawal, with limited documented counseling on medication-assisted treatment, even among those with documented AUD. While all three service lines engage in distinct, yet parallel, care, the limited alcohol-related treatment coordination represents a potential gap in protocols. Overall, these findings revealed lower than expected rates of alcohol-specific assessment and intervention across social work, trauma psychology, and psychiatry services for legally intoxicated patients.

Our findings reflect the broader challenge of competing clinical demands faced by specialty services involved in trauma care and the need for early integrated alcohol interventions for legally intoxicated trauma patients involved in MVC. Increased risk for PTSD and/or depression seen in these patients highlights the necessity for interdisciplinary treatment models. Emphasis on integrating alcohol and mental health screening and referral protocols in high-acuity settings like trauma centers is driven by increasing recognition from the COT that alcohol use and mental health conditions are highly prevalent in this population and significantly impact both short-term outcomes and long-term recovery [9, 27].

**Fig. 2:**
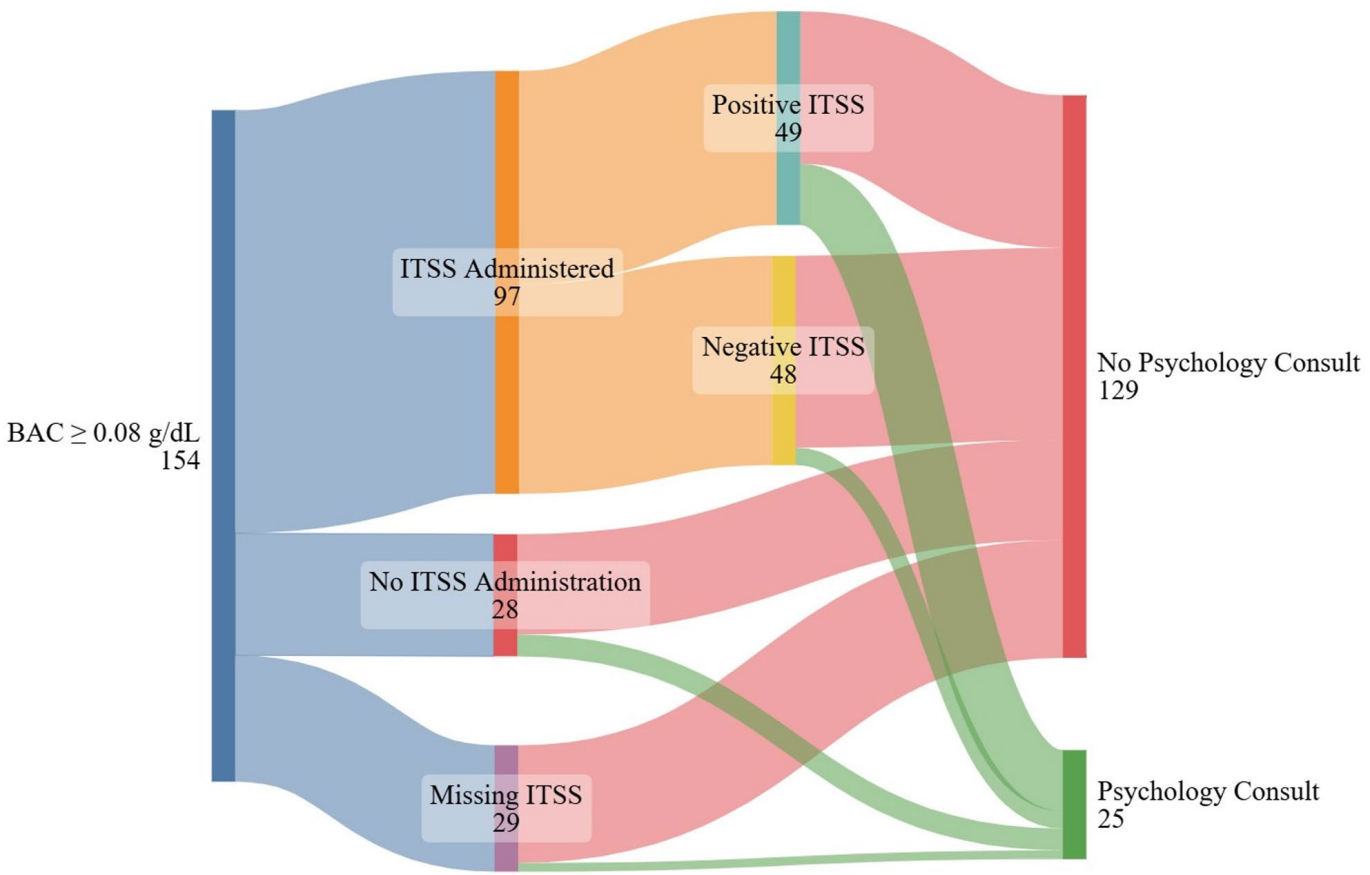
Sankey diagram of ITSS screening and consultation patterns for patients with BAL ≥ 0.08 g/dL

Historically, substance use and mental health concerns have been addressed in sequential treatment models [5, 6]. However, current research suggests the effectiveness of integrated treatment models that concurrently address alcohol use and PTSD and/or depression [20]. This shift represents a greater appreciation of the shared vulnerability and mutual maintenance of these conditions. This is particularly relevant in trauma services as unaddressed PTSD has been associated with increased alcohol misuse and substance related problems among trauma survivors, which may contribute to elevated risk for future alcohol-related injuries [8, 17] Fig 2.

Despite the initial increased burden to implement interventions for co-occurring conditions, the prevention of future alcohol-related injuries reduces the toll on emergency services and trauma care, leading to potential economic [4] and public safety benefits [13]. COT Best Practice Guidelines [ explicitly recommend the inclusion of a dedicated clinician to address alcohol and other substance use concerns, recognizing the competing clinical demands faced by providers.

At this study site, a substance use counselor was hired in 2022 within psychiatry to serve in this dedicated role. One case exemplifies the potential enhancement in care in which a patient with a significant alcohol use history was evaluated by the new dedicated substance use counselor. The documented screening, intervention, and referral processes in this case were substantially more comprehensive, spanning both psychopharmacological and behavioral domains, demonstrating their domain-specific expertise. This example underscores the potential benefits of institutional investment in dedicated personnel and integrated programming to ensure that the complex needs of patients’ high-risk comorbidities are adequately addressed. However, this was not representative of the majority of patients’ experiences and likely requires greater implementation efforts. These findings highlight the critical importance of moving beyond the current standard of parallel treatments and advancing toward fully integrated models of care.

Screening and referral protocols for alcohol use and mental health risk have grown slowly and independently over time [7]. This staggered development and decentralization of implementation has likely contributed to siloed intervention protocols. While this approach meets institutional requirements, it does not always facilitate the delivery of fully integrated, comprehensive care, even within the context of interdisciplinary treatment teams.

Findings from this study illustrate this challenge. The institution examined had long-established alcohol screening and referral protocols in place, as well as integrated psychology services.

However, notable gaps emerged in the management of patients presenting with co-occurring alcohol use and mental health concerns during their hospitalization. This underscores the importance of ongoing efforts to evaluate the implementation of such services, thereby continually improving treatment and addressing potential gaps in care.

Our findings are consistent with established best practice guidelines [1] and the existing literature emphasizing the critical importance of integrated services to address the complex needs of trauma patients with co-occurring mental health and substance use concerns [13, 29]. In alignment with recommendations from the [1], our results highlight the need for systematic processes to ensure BAC assessment for all MVC patients as a key component of SBIRT protocols, enhancing both identification and intervention for patients with alcohol-related concerns. Integrated psychological services should receive targeted training in brief alcohol-related interventions appropriate for hospital settings to facilitate the incorporation of alcohol use assessment and intervention as a standard component of psychological consultation. Increased emphasis on interdisciplinary collaboration is also warranted, as greater integration among hospital-based services providing alcohol-related interventions may enhance the scope and depth of psychosocial intervention during admission and promote the development of comprehensive referral pathways for patients with co-occurring mental health and alcohol-related concerns. In this study, nearly half of the referrals to integrated psychological services originated directly from the trauma team. Trauma centers can leverage interdisciplinary relationships and provide additional training for trauma services, emergency medicine, and social work departments to strengthen identification and referral processes, thereby improving access to hospital-based psychological and psychiatric care for alcohol use assessment and intervention.

### Limitations and future directions

This study was conducted at a single Level I trauma center with a relatively small subsample of legally intoxicated patients receiving trauma psychology consultation. These findings may inform interdisciplinary collaboration at trauma centers with behavioral health teams, however, may not generalize to trauma centers, geographic regions, or patient populations with different behavioral health service models. Given this was a retrospective chart review, the design inherently limits data accuracy to what was documented in the medical record.

Documentation of alcohol screening, intervention content, and psychological symptoms may vary due to competing clinical priorities and the need to balance comprehensive documentation with patient confidentiality. As a result, some aspects of service delivery may not have been fully captured in the medical record. Further, chart abstraction relied on qualitative coding of clinical notes across multiple providers and disciplines. Despite the use of a structured abstraction template, differences in provider documentation style, terminology, and clinical focus may have introduced variability in coding and interpretation.

Due to the inclusion requirements, an MVC as the mechanism of injury, a documented BAC ≥ 0.08 g/dL, and receipt of a psychology consult, the sample may reflect a subgroup of patients with higher clinical visibility or greater engagement with care. As a result, findings may not represent all intoxicated trauma patients, particularly those who were not screened or declined services. This study was further limited by a focused view on inpatient data and did not include follow-up assessments to evaluate post-discharge engagement with treatment, recurrence of alcohol use, or mental health outcomes. Future studies should explore the long-term impact of consultation and referral practices on recovery.

## Conclusion and future directions

This study highlights the overlap in alcohol intoxication and psychological risk among those hospitalized following MVC. Further, this study identified gaps in the processes of addressing these co-occurring concerns. Across the specialty services that engage with patients, alcohol-related assessment and intervention were inconsistently documented, and coordination of treatment appeared limited. Based on the data evaluated in this study, cross-collaboration is vital among social work, psychiatry, and psychology, and models of care should proactively address the potential gaps in patient care for those at risk. Furthermore, this data serves as a call to action to ensure that interdisciplinary treatment teams have clearly defined roles, especially in high-risk patient encounters. These findings suggest the current model of parallel service delivery may not fully address the multifaceted needs of this patient population.

By strengthening assessment and intervention for patients seriously injured in alcohol-impaired MVCs, multidisciplinary teams have a critical opportunity to improve outcomes and reduce the risk of re-injury. Institutional investment in dedicated substance use personnel and having established care pathways may further aid in comprehensive intervention. Beyond individual care, coordinated integrated models of care can also help mitigate future alcohol-related injuries and other public health impacts of impaired driving. Continued evaluation of implementation practices across systems will ensure the best practice guidelines translate into high-quality care for this vulnerable patient population.

Future studies may benefit from examining the role of having a dedicated substance use provider within a trauma system and studying whether this model improves treatment engagement following MVC-related injuries, as well as other mechanisms of injury. Lastly, standardizing documentation practices and establishing integrated care pathways may optimize interdisciplinary collaboration and improve continuity of care for these high-risk patients.

## Data Availability

No datasets were generated or analysed during the current study.

## References

[1] American College of Surgeons. (2023). Best practice guidelines: Screening and intervention for mental health disorders and substance use and misuse in the acute trauma patient. https://www.facs.org/media/nrcj31ku/mental-health-guidelines.pdf 10.1016/j.jen.2023.03.001 37393072

[2] American College of Surgeons Committee on Trauma. Resources for Optimal Care of the Injured Patient; 2020.

[3] Babor TF, Casswell S, Graham K, Huckle T, Livingston M, Rehm J, Room R, Rossow I, Sornpaisarn B. Alcohol: No ordinary commodity-A summary of the third edition. Addiction (Abingdon England). 2022;117(12):3024–36. 10.1111/add.16003. 36321607 10.1111/add.16003

[4] Barbosa C, Cowell A, Bray J, Aldridge A. The cost-effectiveness of alcohol screening, brief intervention, and referral to treatment (SBIRT) in emergency and outpatient medical settings. J Subst Abuse Treat. 2015;53:1–8. 10.1016/j.jsat.2015.01.003. 25648375 10.1016/j.jsat.2015.01.003

[5] Baslock D, Manuel JI, Stanhope V. Incentivizing co-occurring disorder diagnoses through blended payments. Soc Sci Med. 2026;389:118849. 10.1016/j.socscimed.2025.118849. 41338045 10.1016/j.socscimed.2025.118849 PMC12964388

[6] Bruns DP, Kraguljac NV. Co-occurring opioid use disorder and serious mental illness: A selective literature review. J Nurs Scholarsh. 2023;55(3):646–54. 10.1111/jnu.12879. 36734070 10.1111/jnu.12879

[7] Bulger EM, Johnson P, Parker L, Moloney KE, Roberts MK, Vaziri N, Seo S, Nehra D, Thomas P, Zatzick D. Nationwide survey of trauma center screening and intervention practices for posttraumatic stress disorder, firearm violence, mental health, and substance use disorders. J Am Coll Surg. 2022;234(3):274–87. 10.1097/XCS.0000000000000064. 35213489 10.1097/XCS.0000000000000064 PMC10234338

[8] Fendrich M, Petranu K, Nickel L, Larson CL, deRoon-Cassini TA. PTSD symptoms and substance use problems in traumatic injury patients: A 24-month follow-up. Addict Behav. 2026;175:108596. 10.1016/j.addbeh.2026.108596. 41605128 10.1016/j.addbeh.2026.108596 PMC13626385

[9] Gentilello LM, Donovan DM, Dunn CW, Rivara FP. Alcohol interventions in trauma centers: Current practice and future directions. JAMA. 1995;74(13):1043–8. 10.1001/jama.1995.03530130049027. 7563455

[10] Gentilello LM. Alcohol and injury: American college of surgeons committee on trauma requirements for trauma center intervention. J Trauma: Injury Infect Crit Care. 2007;62(6):S44–5. 10.1097/TA.0b013e3180654678. 10.1097/TA.0b013e3180654678 17556967

[11] Hruska B, Pacella ML, George RL, Delahanty DL. The association between daily PTSD symptom severity and alcohol-related outcomes in recent traumatic injury victims. Psychol Addict Behav. 2017;31(3):326–35. 10.1037/adb0000262. 28263624 10.1037/adb0000262

[12] Hunt JC, Sapp M, Walker C, Warren AM, Brasel K, deRoon-Cassini TA. Utility of the injured trauma survivor screen to predict PTSD and depression during hospital admission. J Trauma Acute Care Surg. 2017;82(1):93–101. 10.1097/TA.0000000000001306. 27787440 10.1097/TA.0000000000001306

[13] Kodadek LM, Freeman JJ, Tiwary D, Drake M, Schroeder EM, Rattan R. Alcohol-related trauma reinjury prevention with hospital-based screening in adult populations. J Trauma. 2020;88(1):106–12. 10.1097/TA.0000000000002501. 10.1097/TA.0000000000002501 31490336

[14] Love J, Zatzick D. Screening and intervention for comorbid substance disorders, PTSD, depression, and suicide: A trauma center survey. Psychiatric Serv. 2014;65(7):918–23. 10.1176/appi.ps.201300399. 10.1176/appi.ps.201300399 PMC4256134 24733143

[15] MacLeod JBA, Hungerford DW. Alcohol-related injury visits: Do we know the true prevalence in U.S. trauma centres? Injury. 2011;42(9):922–6. 10.1016/j.injury.2010.01.098. 22081821 10.1016/j.injury.2010.01.098

[16] McBain SA, Sexton KW, Palmer BE, Landes SJ. Barriers to and facilitators of a screening procedure for PTSD risk in a level I trauma center. Trauma Surg Acute Care Open. 2019;4(1):e000345. 10.1136/tsaco-2019-000345. 31467988 10.1136/tsaco-2019-000345 PMC6699788

[17] Nguyen J, Whiteside LK, Bulger EM, Veach L, Moloney K, Russo J, Nehra D, Wang J, Zatzick DF. Post-traumatic stress disorder (PTSD) symptoms and alcohol and drug use comorbidity at 25 US level I trauma centers. Trauma Surg Acute Care Open. 2022;7(1):e000913. 10.1136/tsaco-2022-000913. 35979039 10.1136/tsaco-2022-000913 PMC9358953

[18] Nunn J, Erdogan M, Green RS. The prevalence of alcohol-related trauma recidivism: A systematic review. Injury. 2016;47(3):551–8. 10.1016/j.injury.2016.01.008. 26830122 10.1016/j.injury.2016.01.008

[19] Patarino M, Sanders J, Schindler AG. Mechanisms underlying hazardous alcohol use after mild traumatic brain injury. Alcohol Res. 2025;45(1):09. 10.35946/arcr.v45.1.09. 40917501 10.35946/arcr.v45.1.09 PMC12413194

[20] Persson A, Axén Å, Capusan AJ, Magnusson Å, Heilig M. Concurrent treatment of posttraumatic stress disorder and alcohol use disorder in women: A randomized clinical trial. JAMA Netw Open. 2025;8(7):e2521087. 10.1001/jamanetworkopen.2025.21087. 40663349 10.1001/jamanetworkopen.2025.21087 PMC12371515

[21] Pozzato I, Craig A, Gopinath B, Kifley A, Tran Y, Jagnoor J, Cameron ID. Outcomes after traffic injury: mental health comorbidity and relationship with pain interference. BMC Psychiatry. 2020;20(1):189. 10.1186/s12888-020-02601-4. 32345257 10.1186/s12888-020-02601-4 PMC7189452

[22] Siems A, Banks R, Holubkov R, Meert KL, Bauerfeld C, Beyda D, Berg RA, Bulut Y, Burd RS, Carcillo J, Dean JM, Gradidge E, Hall MW, McQuillen PS, Mourani PM, Newth CJL, Notterman DA, Priestley MA, Sapru A, Wessel DL, Pollack MM. Structured chart review: Assessment of a structured chart review methodology. Hosp Pediatr. 2020;10(1):61–9. 10.1542/hpeds.2019-0225. 31879317 10.1542/hpeds.2019-0225 PMC6931034

[23] Sullivan JT, Sykora K, Schneiderman J, Naranjo CA, Sellers EM. Assessment of alcohol withdrawal: The revised clinical institute withdrawal assessment for alcohol scale (CIWA-Ar). Br J Addict. 1989;84(11):1353–7. 10.1111/j.1360-0443.1989.tb00737.x. 2597811 10.1111/j.1360-0443.1989.tb00737.x

[24] Wan JJ, Morabito DJ, Khaw L, Knudson MM, Dicker RA. Mental illness as an independent risk factor for unintentional injury and injury recidivism. J Trauma: Injury Infect Crit Care. 2006;61(6):1299–304. 10.1097/01.ta.0000240460.35245.1a. 10.1097/01.ta.0000240460.35245.1a 17159669

[25] Wisconsin Department of Transportation. (n.d.). Drunk driving law. Wisconsin.gov. Retrieved from: https://wisconsindot.gov/Pages/safety/education/drunk-drv/ddlaw.aspx

[26] Won NY, McCabe AJ, Cottler LB. Alcohol-related non-fatal motor vehicle crash injury in the US from 2019 to 2022. Am J Drug Alcohol Abus. 2024;50(2):252–60. 10.1080/00952990.2024.2309336. 10.1080/00952990.2024.2309336 PMC11818345 38488589

[27] Yaw LK, Burrell M, Ho KM. Long-term outcomes and determinants of new-onset mental health conditions After trauma. JAMA Netw Open. 2025;8(3):e250349. 10.1001/jamanetworkopen.2025.0349. 40063026 10.1001/jamanetworkopen.2025.0349 PMC11894494

[28] Zatzick DF, Rowhani-Rahbar A, Wang J, Russo J, Darnell D, Ingraham L, Whiteside LK, Guiney R, Hedrick MK, Rivara FP. The cumulative burden of mental, substance use, and general medical disorders and rehospitalization and mortality after an injury. Psychiatric Serv. 2017;68(6):596–602. 10.1176/appi.ps.201600311. 10.1176/appi.ps.201600311 PMC5550030 28142384

[29] Zimmermann E, Sample JM, Zimmermann ME, Sullivan F, Stankiewicz S, Saldinger P. Successful implementation of an alcohol screening, brief intervention, and referral to treatment program. J Trauma Nurs. 2018;25(3):196–200. 10.1097/JTN.0000000000000368. 29742634 10.1097/JTN.0000000000000368

[30] Ziobrowski HN, Holt-Gosselin B, Petukhova MV, King AJ, Lee S, House SL, Beaudoin FL, An X, Stevens JS, Zeng D, Neylan TC, Clifford GD, Linnstaedt SD, Germine LT, Bollen KA, Rauch SL, Haran JP, Storrow AB, Lewandowski C, Musey PI, Kessler RC. Childhood adversities and risk of posttraumatic stress disorder and major depression following a motor vehicle collision in adulthood. Epidemiol Psychiatric Sci. 2023;32. 10.1017/S2045796022000798. 10.1017/S2045796022000798 PMC9879881 36624694

